# Using Resident Health Advocates to Improve Public Health Screening and Follow-Up Among Public Housing Residents, Boston, 2007-2008

**Published:** 2010-12-15

**Authors:** Jo-Anna L. Rorie, Adriana Smith, Tegan Evans, C. Robert Horsburgh, Daniel R. Brooks, Rachel Goodman, Doris Bunte, Lee Strunin, Daisy de la Rosa, Alan Geller

**Affiliations:** Department of Community Health Sciences, Boston University School of Public Health; Boston University Goldman School of Dental Medicine, Division of Community Health Programs, Boston Massachusetts; Boston University Goldman School of Dental Medicine, Division of Community Health Programs, Boston Massachusetts; Boston University Goldman School of Dental Medicine, Division of Community Health Programs, Boston Massachusetts; Boston University Goldman School of Dental Medicine, Division of Community Health Programs, Boston Massachusetts; Boston University Goldman School of Dental Medicine, Division of Community Health Programs, Boston Massachusetts; Boston University Goldman School of Dental Medicine, Division of Community Health Programs, Boston Massachusetts; Boston University Goldman School of Dental Medicine, Division of Community Health Programs, Boston Massachusetts; Boston University Goldman School of Dental Medicine, Division of Community Health Programs, Boston Massachusetts; Boston University Goldman School of Dental Medicine, Division of Community Health Programs, Boston Massachusetts

## Abstract

**Introduction:**

Promoting screening for hypertension, high cholesterol, diabetes, and dental disease, particularly among residents of public housing, is a key strategy for achieving the objectives of *Healthy People 2010*. This community-based participatory research study tested a resident health advocate (RHA) intervention in public housing to increase use of mobile screening and to assess postscreening follow-up care for people with positive screening results.

**Methods:**

During the summers of 2007 and 2008, a mobile health unit screened residents at 4 housing developments for hypertension, high cholesterol, diabetes risk, and dental disease. In the first summer, at 2 intervention sites, RHAs used personal contacts and repeated flyers to recruit residents; 2 control sites received standard recruitment, which was to leave flyers with the development manager. In the second summer, the 2 control sites from the previous year became intervention sites. For both summers combined, we calculated the number of people at intervention and control sites who used the van and we examined rates of appointments made and kept for residents who had positive screening test results.

**Results:**

Screening rates were higher in the intervention condition compared with the control condition (relative risk [RR], 1.55; 95% confidence interval [CI], 1.12-2.15). Approximately 65% of participants screened positive for at least 1 condition. The proportion of participants with screen-positive findings who had follow-up appointments increased from 15% in 2007 to 55% in 2008.

**Conclusion:**

The use of RHAs increased participation in health screening among public housing residents and rates of follow-up medical visits for people with positive screening results.

## Introduction

Residents of government-subsidized public housing have high rates of poor health outcomes (1,2). A health survey of Boston, Massachusetts, residents found that public housing residents reported poorer health than other city residents (3). More than one-third of public housing residents reported being diagnosed with hypertension, more than one-fourth had high cholesterol, and approximately one-fourth of residents were missing 6 or more teeth. Use of dental services was suboptimal; 28% of respondents had not had their teeth cleaned in more than 2 years (4).

Increasing screening for diabetes, hypertension, high cholesterol, and dental disease is a key strategy for achieving the objectives of *Healthy People 2010* (5). Since 2000, the Boston Public Health Commission's (BPHC's) mobile van has offered health screenings to residents of Boston-area public housing developments, but screenings have been underused (G. Thomas, BPHC, November 2008). New motivational and recruitment strategies are needed to increase use.

We decided to use resident health advocates (RHAs) to address gaps in screening services for public housing residents on the basis of proven health education strategies observed in other settings (6,7). Health advocates such as RHAs have been used in many health promotion roles and settings: to provide psychosocial support, to develop relationships with the community and service providers, to assist with insurance, to increase health awareness, and to discuss screening and diagnostic procedures with patients before they receive the services (8,9). Many barriers can be overcome through the use of RHAs, who are viewed by community members as credible sources and who have special knowledge and understanding of perspectives of program participants that professional counselors may lack. The health advocate model has been used in public housing and similar settings for various health conditions and interventions, including asthma, smoking cessation, diabetes, and cancer screenings, and randomized trials with the model have shown improvements in health outcomes (10-14). A literature review of 13 outcome effectiveness studies found that the evidence for community health worker effectiveness was strongest in regard to these workers' impact in increasing access to care (15).

This research study was the result of 5 years of collaboration between the Partners in Health and Housing Prevention Research Center (PHH-PRC) partners. Residents and their representatives were integral participants in the conception, planning, execution, and analysis of the research, which followed community-based participatory research (CBPR) principles (16). The study had 2 objectives: 1) to determine whether RHAs could increase the proportion of public housing residents who received health  screenings at a mobile unit for 4 of the most common chronic conditions: hypertension, high cholesterol, diabetes, and dental disease, and 2) to assess postscreening follow-up care at community health centers.

## Methods

### Sample

In 2007, nine of the 26 family developments managed by the Boston Housing Authority (BHA) had an active RHA. Four housing developments were selected for this study on the basis of the following criteria: presence or absence of an RHA, community health center within 1 mile, and availability of an adequate parking space for the van. Two intervention sites were selected and pair-matched with a control site that had a similar number of adults aged 18 years or older, proximity to a community health center, location, and previous mobile health screening visits (Table 1). In 2008, the 2 sites that did not have RHAs serving as controls in 2007 received the intervention.

The Boston University institutional review board approved the research design, all data collection instruments, and consent forms. For the first study outcome, van use and screening results, informed consent was not required because mobile health screening was considered a standard community service, particularly among residents of public housing. For the second outcome, verification of an appointment, we sought screening participants' consent and personal information to follow up with health centers to verify whether appointments were kept.

### Resident health advocates

The PHH-PRC has trained RHAs every year since 2002 to improve the health status of BHA residents. RHAs are recruited to the training program through an application process. Twelve applicants are chosen each year for a 14-week training program (6 hours per week). The BHA then hires RHAs to work 6 hours per week at their developments, where they educate their fellow residents and become a health information resource for their communities. Each RHA identifies another resident (designated a "peer leader") to assist in distributing flyers and recruiting housing residents on mobile health screening days.

### Recruitment

The primary task of the RHAs in our study was to use various outreach strategies to motivate residents aged 18 years or older to attend mobile health screenings. Before the arrival of the screening van in 2007, RHAs and peer leaders, via one-to-one conversations, encouraged residents at the 2 intervention sites to use the mobile health screening service to address concerns regarding screening. One-to-one conversations took place in various settings (eg, in the management office, at the tenant task force meetings, in the hallways, in parking lots). More than 3,000 flyers in English and Spanish were distributed door-to-door to notify the 1,715 residents of screening dates and times for June through August 2007 and to provide specific health information. Residents of control sites received the same flyer, which tenant management staff distributed in accordance with earlier BPHC protocols.

In 2008, we first conducted a door-to-door survey to determine optimal venues for distribution of flyers and appropriate messages for the flyers. Survey responses led us to create a new, more visually appealing and easier-to-read flyer. Recruitment flyers were also distributed in new locations such as laundry rooms, mail stops, and rooms where bingo games were held.

### The mobile health screening service

The BPHC's mobile public health van, "The Health Connection," is a medical mobile unit that has provided residents of Boston's neighborhoods with free on-site health education and health promotion screening services since 2000. During the summer of 2007, the mobile health screening service came 3 times to each of the 4 sites, for a total of 9 hours per site. Rotating morning, afternoon, and evening schedules were held constant at intervention and control sites. At intervention sites, 5 to 8 RHAs and peer leaders including at least 1 bilingual RHA helped residents to the van, informed them of the pilot study, and processed referral information for those who agreed to participate in the pilot study. We reviewed screening data from 2007 and found that most people visited the mobile unit during afternoon and evening sessions. Consequently, in 2008, the mobile unit conducted afternoon and evening visits only. During the summers of 2007 and 2008, dental examiners worked in the van during 3 of the 6 visits at both intervention and control sites. In 2008, they were present for 4 of the 6 intervention visits.

### Measures

All participants were asked to complete an intake form before receiving screening services. The intake form asked for demographic information, health and health care access information, how they heard about the van, whether they had been screened before, and timing of the last visit to a doctor. We defined "untreated positive screen" as a screen-positive result in a participant who was not previously aware of his or her condition or who had not been taking a prescribed medication for the condition or both.

### Screening tests and activities

Activities described in this section apply equally to participants at both intervention and control sites as part of the standard care offered by the public health mobile unit. Participants were screened for hypertension, high cholesterol, glucose, diabetes risk, and dental disease. Blood pressure and cholesterol cutoffs were those endorsed by the US Preventive Services Task Force (Box) (17). To assess diabetes risk, we used a standard 8-question self-administered tool that asked about diet, exercise, age, and diabetes in the family. Scores could range between 0 and 2 (very low risk), 3 and 9 (low to medium risk), and 10 or more (high risk) (18). High-risk participants were offered a blood glucose screening test. Results were categorized as either normal (mean plasma glucose level ≤140 mg/dL) or high (>140 mg/dL). Dental screening consisted of a visual and tactile inspection of the gums, teeth, and tongue and other soft tissues for irregularities or sensitivity (19). A dental examiner from Boston University Goldman School of Dental Medicine joined the mobile unit health staff to provide dental screenings for presence of root tips and untreated caries, number of missing teeth, denture stability and retention, and other soft tissue abnormalities. Any inspection that resulted in a referral for follow-up care was considered a positive screen.

**Box.** Measures for Blood Pressure, Cholesterol, and Glucose Levels; Diabetes Risk; and Dental Score, Mobile Health  Screening Intervention Using Resident Health Advocates, Boston, 2007-2008

**Blood pressure, systolic/diastolic (mm Hg)^a^ **	
Normal	<120/<80
Prehypertension	120-139 or 80-89
Hypertension, Stage 1^b^	140-159 or 90-99
Hypertension, Stage ≥2 b	≥160 or ≥100
**Cholesterol (mg/dL)^a^**	
Normal	<200
Borderline^b^	200-240
High^b^	>240
**Glucose level (mg/dL)^c^**	
Normal	≤140
High^b^	>140
**Diabetes risk score^c^**	
0-2	very low risk
3-9	low to medium risk
≥10^b^	high risk
**Dental score^d^**	
0	no obvious problems
1	no referral, nonurgent
2^b^	referral, nonurgent
3^b^	urgent dental care within 24 hrs

a US Preventive Services Task Force (17).

b Screen-positive result.

c Heikes et al (18).

d Association of State and Territorial Dental Directors (19).

### Pilot process to increase postscreening follow-up care to community health centers

In 2007, residents with screen-positive results were offered help in making an appointment at the health center of their choice at both intervention and control sites. At both intervention and control sites, RHAs made appointments either on-site or at a later time, in which case the person being referred was called with the appointment information.

On the basis of our experience with referrals in 2007, we pilot-tested a process in 2008 that involved several steps to improve appointment-making and to ensure that appointments were kept. First, we reduced to 2 pages the consent form requesting participants' permission for research staff to seek follow-up information. The revised medical intake form also included 2 new boxes to record whether the participant needed a referral and if the appointment was urgent. Van visits were scheduled for the time of day when health centers were more accessible for appointment-making (usually midday). RHAs also accompanied people to their appointments, translated as needed, and offered information on services for the uninsured.

### Data analysis

We entered data on demographics, access to health care, health information, and screening results from the mobile health unit intake forms and referral forms into an Excel 2003 spreadsheet (Microsoft Corporation, Redmond, Washington) and conducted statistical analysis with SAS  version 9.1 (SAS Institute, Inc, Cary, North Carolina). The primary analysis compared use of the mobile health screening service in the intervention with control conditions. The 2 control housing developments in 2007 constituted the control condition; the intervention condition included the experience at the intervention sites in 2007 in addition to the 2008 experience at the housing developments that had been control sites in 2007. The use of the mobile health screening service was measured as the proportion of the total adult population (aged ≥18) who received screening services. From BHA records, we obtained the number of adult residents by sex, age, race/ethnicity, and primary language spoken at home.

We calculated relative risk (RR) for participants' attendance with 95% confidence intervals (CIs) to compare the proportion of intervention and control condition residents who attended screenings. We calculated *P* values by using χ^2^ or *t* tests as appropriate. Because there were no morning screenings in 2008 and, therefore, more afternoon and evening visits in the intervention condition, we calculated a standardized RR, weighted by the total number of morning (n = 4), afternoon (n = 8), and evening (n = 6) sessions in 2007 and 2008. We also compared the distribution of participants at intervention and control sites in 2007 by sex, age, race/ethnicity, time of screening (morning, afternoon, or evening), primary language spoken at home, education, medical and dental insurance status, and most recent primary care visit.

To assess the role of RHAs in facilitating follow-up medical care after a positive screening, we compared the proportion of consent forms completed (to gauge the success of the enrollment process) and the proportions of appointments made and kept in the summer of 2007 with those in the summer of 2008. We combined intervention and control sites in 2007 because there were no differences in the nature or intensity of efforts to increase follow-up at either set of sites.

## Results

### Screening study

In 2007, 6% (n = 100) of adult residents at the 2 intervention sites were screened, compared with 3% (n = 47) of residents from control sites (RR, 1.74; 95% CI, 1.24-2.44). Use at the intervention sites, adjusted for time of day of mobile health screening visits, was also higher than at control sites for both years combined (RR, 1.55; 95% CI, 1.12-2.15).

For both intervention and control sites combined in 2007, mobile health screening participants were primarily female, Hispanic, had a mean age of 44, and had completed high school (Table 2). Hispanic (57% of mobile health screening participants compared with 41% of adult residents, *P* < .001) and male residents (39% of mobile health screening participants compared with 27% of adult residents, *P* = .001) used the van at rates disproportionate to their numbers in the developments.

Of the 224 participants from intervention and control sites across both years, 146 (65%) had at least 1 positive screening diagnosis for hypertension, high cholesterol, diabetes risk, or dental disease. Of the 217 participants screened for hypertension, 64 had either stage 1 (n = 36) or stage 2 (n = 28) hypertension. Twenty of the 64 participants with stage 1 or stage 2 hypertension had not seen a doctor in the past 12 months, and 39 were considered an untreated positive screen (Table 3).

Although 25% of participants had a screening diagnosis of hypertension and 24% had high cholesterol, findings of diabetes risk and dental disease were more common. Two-thirds of those screened (n = 114) had a high diabetes risk score based on the 8-question self-administered tool, and 9 of the 114 had a positive blood glucose test (Table 3). A total of 127 people attended the mobile health screening service on days when screening for dental disease was provided, 49 of whom chose not to use dental services. Of the 78 who were screened, 41 (53%) were referred to a dentist for follow-up. Referrals were based on a 0-3 scoring system used by the dental staff (Box). Urgent care was recommended for 3 participants who reported significant pain or had obvious infection.

### Pilot study to increase postscreening follow-up care

In 2007, among the 91 participants who screened positive for any condition, 44 (48%) provided consent for follow-up. Of these, appointments were made for 27 participants (61%) within 3 months after the date of the screen-positive finding. Appointments were not made for 17 participants for 2 major reasons: disconnected telephone lines or inability to contact the resident after several telephone calls (n = 8) and system barriers (n = 9). Fourteen (52%) of the 27 participants with appointments kept them. In 2008, among the 44 participants who screened positive, 33 (75%) provided consent for follow-up. Of those who consented, appointments were made for all 33 participants within 1 month after the date of the screen-positive finding. Of the 33 participants with appointments, 24 (73%) had kept an appointment within 2 months after the initial screening. Overall, the proportion of all screen-positive participants who completed a follow-up medical appointment through the mobile health screening mechanism increased from 15% (14 of 91) in 2007 to 55% (24 of 44) in 2008.

## Discussion

Community-based interventions are most successful when they are designed with communities as respected partners, address problems in the context of community strengths, respect cultural diversity, and use outreach workers (20-22). Three previous studies have reported success in the use of RHAs in low-income and senior citizen housing to boost health promotion practices related to smoking cessation (10), prevention of HIV infection (21,23), and mammography (24).

In our study, we recruited and trained public housing residents as RHAs to motivate other public housing residents to use a city-run mobile public health unit for health screening. The results of the intervention showed that intervention sites with RHAs had higher rates of screening for chronic diseases on the mobile health screening service compared with control sites with no RHAs. Although we cannot isolate individual recruitment strategies that may have been the most responsible, RHAs actively and frequently distributed colorful, bilingual flyers at intervention sites; recruited fellow residents as peer leaders; provided one-to-one motivational advice; and were accessible to fellow residents.

In previous studies, outreach workers were associated with significant increases in cervical cancer screening (14), mammography education (24), and diabetes education (12). The magnitudes of these increases were 17%, 40%, and 70%, respectively. Such results are similar in magnitude to the results obtained in this study, in which medical visits increased to 55% (a relative difference of 72%) among populations receiving the RHA-delivered screening promotion message.

Although the RHA intervention increased use of on-site van screening services, participants represented only about 6% of adults at the intervention developments. On average, about 5 people per hour were screened, well below the potential capacity of 10 to 12 visits per hour. However, our intervention was limited to one 4-hour visit per month to each development for 3 successive months. We believe that additional visits would have increased use and that continued presence of the van would have resulted in van use by a substantial number of residents.

During the first summer, only 15% of residents with screen-positive findings had a follow-up medical appointment. We identified important barriers and, before the next summer, we developed training programs, an expedited referral process, and new intake forms. We scheduled van visits to coincide with better calling hours to health centers and added a component in which RHAs accompanied residents to follow-up appointments. As a result, compared with the first summer, rates of appointments kept increased more than threefold. Furthermore, all 3 individual components (consent provided, appointments made, and appointments kept) contributed to the overall increase.

This study has a number of limitations. Although the intervention and control sites in 2007 were pair-matched on the basis of location, size, and accessibility to a community health center, each development maintains a unique set of characteristics that makes comparison of sites difficult. Second, the personality and social networks of the individual RHAs may have been an important factor in the successful dissemination of the screening message, but we could not assess these variables. Third, we did not track subsequent use of medical care among attendees who had a screen-positive result if they did not consent or were lost to follow-up in the first year. As a result, we may have overestimated the effect of RHA referral activities. However, the size of the increase in completed appointments from the first to second year was so large that some improvement can probably be attributed to the efforts of the RHAs. Finally, we did not conduct a systematic evaluation of the reasons that residents chose not to participate in the screening, so we do not know the number of residents who should be considered in the target population for screening services nor what barriers prevented their participation.

In conclusion, we found that RHAs significantly increased health screening among residents of Boston public housing developments. In addition, we found high levels of 4 chronic conditions among Boston public housing residents. These high rates underscore the need for expanded screening services, enhanced access to primary care providers, and improved referral networks to treat chronic disease. High rates of self-assessed risk indicate potential benefits of preventive programs for diabetes that use expanded nutritional and exercise counseling. RHA recruitment of fellow residents for screening, if sustained and coupled with clinical follow-up and adherence to medical recommendations, would improve health and would reduce health disparities among public housing residents. Further research is needed to assess whether increases in screening translate into increased clinical follow-up and participation in health-promotion programs.

## Figures and Tables

**Table 1:** Characteristics of Participants by Study Sites, Mobile Health Screening Service Intervention Using Resident Health Advocates, Boston, 2007^a^

Characteristic	Study Site			

	Intervention A,^b^ No. (%)	Intervention B, No. (%)	Control A, No. (%)	Control B, No. (%)
**Age and sex**				
Residents aged ≥18 years	1,181 (100)	534 (100)	1,045 (100)	354 (100)
Women	814 (69)	416 (78)	773 (74)	272 (77)
**Race/ethnicity**				
Black	283 (24)	250 (47)	522 (50)	152 (43)
Hispanic	401 (34)	267 (50)	459 (44)	159 (45)
White	330 (28)	16 (3)	31 (3)	21 (6)
**Language spoken by head of household**				
Primary language Spanish	389 (33)	240 (45)	438 (42)	145 (41)
Primary language English	590 (50)	245 (46)	491 (47)	187 (53)
**Proximity and mobile health screening service history**				
Closest community health center	On-site	<1 mile	On-site	<1 mile
Mobile health screening history	New site	Old site	New site	Old site

a Numbers may not total the number of residents and percentages may not total 100 because missing data were not counted.

b Intervention sites A and B were pair-matched with control sites A and B, respectively.

**Table 2:** Characteristics of Participants and Populations at Intervention and Control Sites, Mobile Health Screening Intervention Using Resident Health Advocates, Boston, 2007^a^

Characteristic	Intervention		Control		*P* Value^b^

	Participants, No. (%), n = 100	Population ≥18 y, No. (%), n = 1,715	Participants, No. (%), n = 47	Population ≥18 years, No. (%), n = 1,399	
Age, mean (SD), y	45.1 (18.7)	NA	42.8 (17.2)	NA	.47
**Sex**					
Women	60 (60)	1,235 (72)	29 (62)	1,049 (75)	.76
Men	40 (40)	480 (28)	18 (38)	350 (25)	
**Race/ethnicity **					
Black	22 (22)	497 (29)	17 (36)	672 (48)	.01
Hispanic	56 (56)	669 (39)	28 (60)	616 (44)	
White	15 (15)	326 (19)	0	56 (4)	
Other	6 (6)	223 (13)	2 (4)	55 (4)	
**Language^c^**					
English	35 (35)	840 (49)	18 (38)	686 (49)	.92
Spanish	57 (57)	617 (36)	26 (55)	588 (42)	
Haitian Creole	4 (4)	NA	1 (2)	NA	
Other/unknown	4 (4)	258 (15)	2 (4)	125 (9)	
**Education **					
Did not attend school	2 (2)	NA	4 (6)	NA	.52
Some primary or secondary	40 (40)	NA	19 (40)	NA	
High school graduate	29 (29)	NA	14 (30)	NA	
Post high school education	29 (29)	NA	10 (21)	NA	
**Health insurance **					
None	30 (30)	NA	10 (21)	NA	.46
State-provided	59 (59)	NA	29 (62)	NA	
Private	11 (11)	NA	8 (17)	NA	
**Dental insurance**					
Yes	57 (57)	NA	28 (60)	NA	.85
No	43 (43)	NA	19 (40)	NA	
**Last visit to doctor**					
<6 months ago	52 (52)	NA	26 (55)	NA	.55
6-11 months ago	22 (22)	NA	6 (13)	NA	
1-2 years ago	16 (16)	NA	8 (17)	NA	
>2 years ago	11 (11)	NA	7 (15)	NA	

Abbreviation: NA, not available or not assessed.

a Percentages may not total 100% because of rounding.

b *P* value represents comparison between participants at intervention and control sites.

c Language for mobile health participants represents participant's first language; language at sites represents household language spoken.

**Table 3:** Participants' Screening Test Results, Mobile Health Screening Intervention Using Resident Health Advocates, Boston, 2008

Chronic Condition	No. With Positive Screening Results	No. Not Previously Aware of Their Condition^a^	**No. With No Recent Medical Care^b^**
**Hypertension:** 217 total screenings			
Stage 1	36	22	10
Stage 2	28	17	10
**Cholesterol:** 200 total screenings			
Borderline	35	27	6
High	12	10	5
**Diabetes:** 172 risk tests^c^, 103 glucose screenings			
High risk^d^	114	88	30
High glucose	9	6	2
**Dental care:** 78 total screenings			
Nonurgent care	41	4	13

a An "untreated positive screen" is a screen-positive result in a participant who was not previously aware of having the condition (undetected) or who had not been taking a prescribed medication for the condition (detected but untreated).

b Participants who had positive screening results for both hypertension and diabetes and had not been seen by a physician in >12 months.

c To assess diabetes risk, we used a standard 8-question self-administered tool that asked about diet, exercise, age, and diabetes in the family (18).

d Participants with a score of 10 or more on the diabetes risk test were considered at high risk and were offered a blood glucose screening test.
